# Iron Deficiency: A New Target for Patients With Heart Failure

**DOI:** 10.3389/fcvm.2021.709872

**Published:** 2021-08-10

**Authors:** Caterina Rizzo, Rosa Carbonara, Roberta Ruggieri, Andrea Passantino, Domenico Scrutinio

**Affiliations:** Department of Cardiology, Istituti Clinici Scientifici Maugeri, IRCCS, Bari, Italy

**Keywords:** chronic heart failure, acute heart failure, iron deficiency, anemia, ferric carboxy maltose, hemoglobin, iron deficiency anemia

## Abstract

Iron deficiency (ID) is one of the most frequent comorbidities in patients with heart failure (HF). ID is estimated to be present in up to 50% of outpatients and is a strong independent predictor of HF outcomes. ID has been shown to reduce quality of life, exercise capacity and survival, in both the presence and absence of anemia. The most recent 2016 guidelines recommend starting replacement treatment at ferritin cutoff value <100 mcg/l or between 100 and 299 mcg/l when the transferrin saturation is <20%. Beyond its effect on hemoglobin, iron plays an important role in oxygen transport and in the metabolism of cardiac and skeletal muscles. Mitochondria are the most important sites of iron utilization and energy production. These factors clearly have roles in the diminished exercise capacity in HF. Oral iron administration is usually the first route used for iron repletion in patients. However, the data from the IRONOUT HF study do not support the use of oral iron supplementation in patients with HF and a reduced ejection fraction, because this treatment does not affect peak VO_2_ (the primary endpoint of the study) or increase serum ferritin levels. The FAIR-HF and CONFIRM-HF studies have shown improvements in symptoms, quality of life and functional capacity in patients with stable, symptomatic, iron-deficient HF after the administration of intravenous iron (i.e., FCM). Moreover, they have shown a decreased risk of first hospitalization for worsening of HF, as later confirmed in a subsequent meta-analysis. In addition, the EFFECT-HF study has shown an improvement in peak oxygen consumption at CPET (a parameter generally considered the gold standard of exercise capacity and a predictor of outcome in HF) in patients randomized to receive ferric carboxymaltose. Finally, the AFFIRM AHF trial evaluating the effects of FCM administration on the outcomes of patients hospitalized for acute HF has found significantly fewer hospital readmissions due to HF among patients treated with FCM rather than placebo.

## Introduction

Heart failure (HF) is a major cause of morbidity and mortality worldwide, and a growing public health problem: the incidence of HF with reduced ejection fraction is 1–2% in the general population and as high as 10% in the population over 65 years of age.

Patients with HF have many comorbidities that affect their quality of life, clinical management and prognosis (1); however, treatment of comorbidities does not always improve patients outcomes; for example, anemia treatment with agents stimulating erythropoiesis have not demonstrated any benefit in HF or sleep related breathing disorders. On the other hand, the HF course can be improved through treatment of iron deficiency (ID) with ferric carboxymaltose (FCM) and of diabetes with SGLT2 inhibitors.

This review describes the prevalence, clinical implications, and possible advantages of treating iron deficiency in HF.

## Definition Of Iron Deficiency

ID is defined as a “health-related condition in which iron availability is insufficient to meet the body's needs and which can be present with or without anemia” (2).

In daily clinical practice, simple tests used for the diagnosis of ID include serum ferritin, serum iron, transferrin and transferrin saturation (TSAT); however, the best cutoff index for ID remains unclear.

It is useful to distinguish absolute ID, in which a real deficit of iron stores exists, and functional deficiency, in which the iron stores are normal, but the transport of iron to target cells is deficient (3). In functional deficiency, the interpretation of serum ferritin levels may be challenging. In the presence of inflammation, ferritin, an acute phase protein, may become elevated; therefore, in patients with HF, in whom low-grade inflammation is present, ferritin dosage may be not suitable to identify ID. Nanas et al. have found that some patients with HF and anemia have ID (as evaluated by bone marrow biopsy) despite having normal serum ferritin levels (4).

The cutoff values at which replacement treatment is recommended, according to the most recent 2016 ESC guidelines, are a ferritin value <100 mcg/l (absolute ID) or is between 100 and 299 mcg/l when the TSAT is <20% (functional ID) (5). Treatment should be considered regardless of the presence of anemia. Although these cutoff values were not previously validated, they have been used in clinical trials in which IV ion supplementation has been found to be clinically successful; consequently, they are now largely accepted.

The gold standard for identifying ID is bone marrow iron staining. Using this method, which requires bone marrow aspiration from the sternum, Grote Beverborg et al. have validated the definition of ID according to biomarkers. Forty-two patients with HF undergoing sternotomy for coronary bypass were studied. Iron staining was performed on bone marrow aspirated from the sternum, and iron stores were accurately measured. The ESC definition of ID had a sensitivity of 94% and a specificity of 72% for identifying ID. A TSAT ≤ 19.8 was the best cutoff, together with serum iron ≤ 13 μmol/L; the areas under the ROC curves were 0.932 a 0.922, respectively (6). These experimental data have confirmed the accuracy of a cutoff of 20% for TSAT, although they prompt the question of whether serum ferritin is a reliable diagnostic marker for ID.

A major limitation of the ESC definition of ID is the exclusion of patients with ferritin >300 μg/ml and TSAT ≤ 20%.

## Prevalence

Because ID is the most widespread nutritional disorder worldwide, its high prevalence in patients with HF is unsurprising. In that population, the ID prevalence is estimated to be between 35 and 55%. The prevalence of ID is higher in women than men, and it increases with worsening of the NYHA functional class (7).

ID is common regardless of the presence of anemia (8).

In patients with acutely decompensated HF, the prevalence is even higher, ranging from 72 to 83% (9). Absolute ID is more prevalent than relative ID in both acute and chronic HF.

The variability among studies may be explained by the different characteristics of the enrolled populations (some studies included only anemic patients) and by the choice of ID definition. When ID was identified by the most reliable method, bone marrow staining, the prevalence has been found to be 40% (6).

## Pathophysiology Of Iron Deficiency

Iron is the most important and abundant trace element in the human body (3–5 g overall), it is absorbed in the gut, delivered to tissues for erythropoiesis or other functions, deposited in reticuloendothelial cells and lost through epithelial desquamation or bleeding (10). Iron accumulation is toxic and can potentially result in the generation of reactive oxidative species (ROS), which can damage all cell components (11).

Physiological iron metabolism involves transferrin and ferritin. Transferrin is a plasma protein that transports iron through the blood circulation. Iron enters cells through transferrin receptor type 1 via receptor-mediated endocytosis; in the cytoplasm, iron is used to synthetize heme-groups, cytochromes and other Fe-dependent proteins involved in oxygen transport and oxidoreductive reactions (12). In contrast, ferritin enables iron storage.

Both ferritin and transferrin also act as “detoxifiers” in cells and vessels, respectively: they bind ferrous iron (Fe^2+^) and thereby prevent its conversion to the ferric isoform (Fe^3+^) and the concomitant reduction of molecular oxygen, thus leading to ROS formation (13).

Iron is fundamental in aerobic metabolism being the key component of hemoglobin and myoglobin, the main proteins for O_2_ transport and accumulation, respectively. Proteins involved in oxidative energy generation contain iron in their prosthetic groups. Oxidoreductive reactions occur in the cytoplasm via the citric acid cycle and in the mitochondria via the respiratory chain—two central pathways in cellular energy generation (10).

In the citric acid cycle, the conversion of citrate to isocitrate involves a chemical reaction mediated by aconitase. This enzyme has a ferro-sulfo (Fe-S) center, which is sensitive to iron levels (14). Low iron levels result in the loss of iron atoms in Fe-S clusters and the stimulation of trasferrin-receptor-1 mRNA transcription (15).

In the respiratory chain, cytochromes and Fe-S proteins serve as electron transporters in the mitochondrial membrane, and both use iron in prosthetic heme-groups or Fe-S cluster proteins (12).

As described above, iron is involved in crucial metabolic cell reactions, owing to its ability to bind oxygen (O_2_).

Iron acts as an electron transporter in key sites of O_2_ utilization, such as the mitochondria, which are abundant in heart tissue and skeletal muscle, and produce energy for contraction. Consequently, metabolism in tissues with high energetic demand is strictly dependent on iron homeostasis. An unfavorable catabolic-anabolic balance with a metabolic switch toward anaerobic glycolysis in muscular tissue is responsible for decreased exercise capacity (12).

Cultured human stem cell-derived cardiomyocytes subjected to ID exhibit diminished basal respiration, owing to respiratory chain dysfunction. In this *in vitro* model, mitochondria show aberrant structures and localization in cells after the activation of a hypoxic response. The final switch from fatty acid oxidation to anaerobic glycolysis can be rescued by iron restoration (16).

In a mouse model, targeted disruption of transferrin receptor-1 in cardiomyocytes induces early lethal cardiomyopathy, owing to mitochondrial failure and dysfunctional mitochondrial clearance, which contribute to a switch toward anaerobic metabolism. This cardiomyopathy might be prevented by aggressive and continuous iron supplementation (17).

### Principal Mechanisms of ID

ID may have a negative effect on the generation of cellular energy, particularly in cells with high metabolic demand. In HF, ID can contribute to a shift toward anaerobic metabolism. Clinically, ferritin and transferrin saturation blood levels provide information about iron homeostasis, because their synthesis is dependent on iron availability.

Absolute ID is clinically defined by a ferritin level <100 ng/ml, which indicates a decrease in total body iron stores. Its etiology is based on interference with iron transport throughout the body. Three main mechanisms underlie the deficiency: *low iron intake*, which depends on the diet; *decreased iron absorption*, which is associated with gastrointestinal abnormalities; and *increased iron loss*, owing to hemorrhages or proteinuria.

High levels of proinflammatory cytokines can trigger “iron trapping” in macrophages, hepatocytes and enterocytes, through the degradation of ferroportin, the transmembrane protein for iron transport outside cells. This mechanism usually protects against microorganisms, which depend on iron availability for survival, and it promotes a *functional state of ID*, thus making iron unusable despite sufficient iron stores. This pathological state is clinically defined by ferritin levels <300 ng/ml with TSAT ≤ 20% (18, 19).

Low TSAT can also be used to identify patients with absolute ID in the case of high levels of ferritin, which usually acts as a non-specifically acute-phase reactant.

In HF, both types of deficiency can occur (18). Renal dysfunction necessitates a low protein-diet with a consequent low iron intake. In renal dysfunction, proteinuria is frequently observed and also involves Fe-proteins. Antiaggregant/anticoagulant therapies are potentially responsible for gastritis or duodenitis, thereby increasing iron loss. Low arterial blood flow or venous blood accumulation in edema conditions characterize different stages of HF or can coexist in the same phase. Consequently, decreased iron absorption through the enteric edematous mucosa is one mechanism underlying ID in HF. The increasing use of proton-pump inhibitors impairs the process of iron absorption, which is optimal at lower pH in its reduced form Fe^2+^. Other medications prescribed for HF can decrease hematopoietic activity, such as angiotensin-converting enzyme inhibitors or carvedilol (13, 18, 19).

A chronic proinflammatory state is present in HF, as confirmed by the high levels of IL-6, TNF-alpha, and INF-gamma found in patients with HF. Inflammation increases the release of hepcidin, a key liver regulatory protein promoting the degradation of the membrane iron exporter ferroportin (11). Furthermore, enhanced expression of pro-inflammatory cytokines in HF has been correlated with inhibition of erythropoiesis, through elevated levels of negative regulators of hematopoietic stem cells (20).

Together, these mechanisms cause low levels of iron, independently of the level of iron stores. Over time, ID can cause anemia, a common comorbidity in patients with HF.

Anemia and ID share common causes (21). Anemia in HF is usually due to multiple factors, including hypoplastic bone marrow (particularly in older people), inadequate erythropoiesis (vitamin deficiency, low erythropoietin levels, and blunted erythropoietin production) or increased blood loss (hemorrhage and frequent venipuncture, as are common in HF) (22). Another possibility for consideration early in HF is pseudo-anemia, which results from fluid retention with increased extracellular volume (20).

## Clinical Implications Of Id

Low levels of iron are independently associated with diminished exercise tolerance in HF, even in the absence of anemia. In patients with HF, ID correlates with a diminished peak oxygen consumption (VO_2_ peak) and an increased ventilatory response (VE/VCO_2_ slope) in cardiopulmonary exercise tests.

In 27 patients, Okonko et al. have found a lower VO_2_ peak measured during maximal cardiopulmonary tests in patients with ID compared with iron-replete patients.

Aerobic capacity was correlated with TSAT and ferritin, independently of NYHA functional class and hemoglobin level. In addition, the ventilatory response to exercise, expressed as the VE/VCO_2_ slope, was correlated with TSAT (23).

In a larger sample, Ebner et al. have found a diminished maximal aerobic capacity in patients with ID without anemia; in patients with both anemia and ID, the capacity was even lower (24).

Beyond its roles in decreasing exercise capacity (25) and quality of life (26), ID has been demonstrated to be a predictor of prognosis for death and hospitalizations (27).

In several survival studies, ID has been associated with an increased rate of hospitalization and mortality regardless of the presence of anemia (9), despite major differences in the methods chosen to evaluate ID in the prognostic studies.

During a 743-day follow-up, in 157 patients with chronic HF and a left ventricular ejection fraction ≤45%, a TSAT ≤ 20% has been found to be an independent predictor of event free survival. Iron-deficient patients had a 3-fold greater risk of death regardless of whether they were anemic. Patients with ID but without anemia had a 2-fold greater risk of mortality (23).

In an international pooled analysis including 1,506 patients with CHF (with both preserved and reduced ejection fraction) from five cohorts, ID has been found to be a strong predictor of mortality. In multivariable hazard models, ID (defined by ESC criteria), but not anemia, has been found to be a strong independent predictor of mortality (hazard ratio 1.42, 95% confidence interval 1.14–1.77, *p* = 0.002). Furthermore, ID has been found to significantly improve risk classification and integrated discrimination when added to a predictive model incorporating established risk factors (8).

In a study population of 387 patients with HF with reduced ejection fraction, both TSAT ≤ 19.8% and serum iron ≤ 13 μmol/l (ID criteria validated by bone marrow staining) have been found to be predictors of death during a 2 year follow-up, whereas isolated low ferritin was not associated with the risk of death (6).

In 2,356 patients with worsening HF, low iron storage (defined by a bone marrow-validated combination of transferrin saturation <20% and a serum ferritin concentration of 128 ng/mL or less) has been associated with HF prognosis. In the same population, defective iron utilization (defined by a TSAT <20% and a serum ferritin concentration >128 ng/mL) did not predict outcome (28).

Recently, Campodonico et al. have reported that patients with a TSAT ≤ 20%, independently of ferritin levels, had poorer prognosis than those with TSAT ≥ 20% during a 2 year follow-up. When patients were dived according to ferritin level, the group with both composite ferritin between 100 and 300 mg/L and TSAT < 20% identified patients with HF with the poorest survival rate (29).

ID is prevalent in patients with acute HF. Among patients hospitalized for acute HF, absolute ID, compared with functional ID and no ID, has been associated with an increased rate of readmission (19.9, 13, and 13.5%, respectively, *P* = 0.005). Absolute ID remained associated with clinical outcome after correction for other variables (30).

However, at odds with these results, ID has not been found to predict mortality or hospitalization after adjustment for comorbidities in a real life population of 1,684 patients with HF (31). According to the authors, the high percentage of patients with a preserved ejection fraction in the study population may account for this finding.

In 165 patients hospitalized for acute HF, ID, defined by low levels of serum hepcidin and high serum soluble transferrin receptors, has been found to be a significant and independent predictor of 12-month mortality (32).

**Figure 1:**
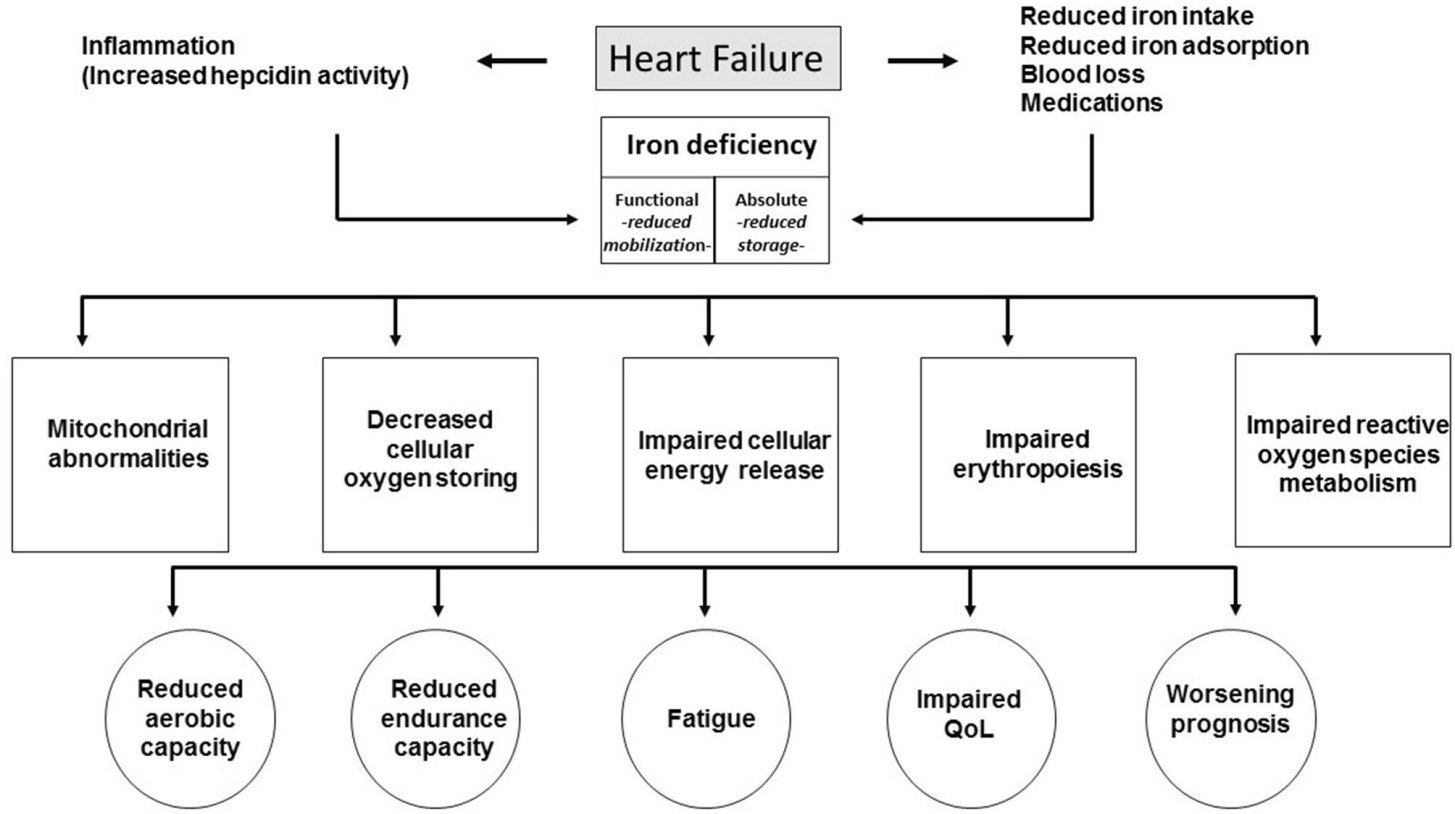
Causes and consequences of ID in HF.

Causes and consequences of ID in HF are summarized in Figure 1.

## Iron Treatment In Patients With Chronic Hf

### Changing the Target: From Anemia to ID

In the past, the treatment of anemia, a relevant comorbidity in HF (33, 34), by increasing red blood cell synthesis through erythropoietin (EPO) administration in HF patients, has been considered a valuable strategy (35–37).

After encouraging results from some preliminary, small studies (38–42), following larger trials, did not demonstrated any clinical improvement after EPO administration. In contrast, an increased risk of ischemic stroke and embolic events was recorded in patients treated with darbepoetin (43–45).

After these disappointing results, ID was identified as a new possible therapeutic target in HF. The main trials with iron supplementation in HF are reported in the Table 1.

**Table 1:** Main trials of iron supplementation in heart failure.

**Trial**	**Pts (n)**	**Design**	**Study drug**	**Inclusion criteria**	**Primary endpoint**	**Results**
IRON-5 HF	54	Double blind randomized controlled	Oral ferrous sulfate 200 mg t.i.d for 90 days	LVEF <50% NYHA II-III (able to perform 6MWT)	Change 6MWT	Terminated early
IRONOUT HF	225	Phase 2 double-blind placebo-controlled randomized	Oral iron polysaccharide 150 mg twice daily for 16 weeks	LVEF ≤ 40% with NYHA II through IV symptoms	Change in peak VO_2_ from baseline to 16 weeks	High-dose of oral iron did not improve exercise capacity over 16 weeks
FERRIC HF	35	Randomized controlled observer-blinded	IV iron sucrose weekly for 16 weeks (Each dose was administered as 200-mg aliquots in 50 ml normal saline infused over 30 min)	CHF (NYHA II or III) with LVEF ≤ 45%	Change in absolute pVO_2_	Intravenous iron loading improved exercise capacity and symptoms
FAIR HF	459	Double-blind placebo-controlled randomized	FCM (intravenous bolus injection of 4 ml) weekly until iron repletion was achieved, then every 4 weeks during the maintenance phase, which started at week 8 or week 12	CHF (NYHA II or III), LVEF of 40% or less (for patients with NYHA II) or 45% or less (for NYHA III)	Self-reported Patient Global Assessment and NYHA functional class at week 24	Intravenous ferric carboxymaltose improves symptoms, functional capacity, and quality of life
CONFIRM HF	304	Double-blind placebo-controlled randomized	FCM solution given as undiluted bolus i.v., injections of 500 or 1,000 mg of iron, administered over at least 1 min	Ambulatory symptomatic HF patients with LVEF ≤ 45%	Change 6 MWT distance from baseline to Week 24	Sustainable improvement in functional capacity, symptoms, and QoL in patients treated with FCM
EFFECT HF	172	Prospective randomized controlled multicenter open-label trial with blinded end-point evaluation	FCM as an undiluted intravenous bolus injection or an infusion of 500 or 1,000 mg administered diluted in ≈100 mL of sodium chloride solution and given in ≥6 min for 10 mL	HFrEF (LVEF ≤ 45%)	Change in peak VO_2_ from baseline to 24 weeks measured by CPET	Favorable effect on peak VO2 was observed in patients treated with FCM.
AFFIRM-AHF	1,132	Double-blind placebo-controlled randomized	FCM administered as an undiluted bolus injection	Patients Admitted for Acute Heart Failure with LVEF <50%	The composite of recurrent HF hospitalizations and CV death up to 52 weeks	Pts actively treated had significantly fewer hospitalization for HF

*Pts, patients; FCM, ferric carboxymaltose; iv, intravenous; LVEF, left ventricular ejection fraction; HFrEF, heart failure with reduced ejection fraction; 6MWT, six minute walking test; CPET, cardiopulmonary exercise test*.

### Oral Iron Supplementation

The Short Term Oral Iron Supplementation in Systolic Heart Failure Patients Suffering From Iron Deficiency Anemia (IRON-5 HF) trial has evaluated the use of oral iron administration. Unfortunately, the trial was terminated early after prolonged recruitment and funding problems.

The Oral Iron Repletion Effects On Oxygen Uptake in Heart Failure (IRONOUT HF) trial, also testing oral iron administration, was a double-blind, randomized, placebo-controlled trial of 225 patients with HF, an LVEF < 40% and ID, defined by a ferritin concentration of 15 to 100 mg/l, or ferritin 101–299 mg/l with TSAT < 20% (46).

The primary endpoint, a change in peak oxygen uptake from baseline to 16 weeks, did not differ between groups at the end of the follow-up. Likewise, no significant changes were found for the secondary endpoints of 6-min walking tests (6MWT) and NT-proBNP levels.

Moreover, oral iron supplementation was limited by collateral effects: approximately 40% of patients receiving oral iron preparations experienced adverse effects, such as nausea, flatulence, abdominal pain, diarrhea, constipation, and black stools.

### Intravenous Iron Supplementation

The Ferric Iron Sucrose in Heart Failure (FERRIC-HF) trial tested the hypothesis that iron repletion alone would improve exercise tolerance in anemic and non-anemic patients with symptomatic CHF and ID. Thirty-five patients with CHF (age 64 ± 13 years, VO_2_ peak 14.0 ± 2.7 ml/kg/min) were randomized to 16 weeks of intravenous iron or no treatment. The ferritin was required to be <100 ng/ml, or 100–300 ng/ml with TSAT <20%. Intravenous iron treatment improved exercise capacity and symptoms. The benefits were more evident in patients with anemia (47).

The Ferinject Assessment in patients with IRon deficiency and chronic Heart failure (FAIR HF) trial was the first large-scale, double-blind, placebo-controlled multicenter trial of FCM in patients with chronic HF (48). The trial recruited 459 patients with HF with reduced ejection fraction, in NYHA functional class II or III, for a follow-up period of 6 months. Patients were required to have ID, defined by serum ferritin <100 mg/l, or ferritin ranging from 100 to 300 mg/l with TSAT < 20%. Patients were randomly allocated in a 2:1 fashion to receive intravenous infusion (IV) of FCM or placebo. The study drug was given in doses based on the participant weight and Hb value at screening. FCM was administered weekly during the correction phase until iron repletion was obtained, then every 4 weeks during the maintenance phase.

The study demonstrated that IV FCM improved the primary endpoint of quality of life, according to the self-reported PGA and NYHA class. At week 24, when primary endpoints were evaluated, in the FCM group, 47% of patients were in NYHA class I or II, as compared with 30% in the placebo group (odds ratio for improvement by one class 2.40; CI 1.55–3.71; *p* < 0.0001).

Secondary endpoints, including PGA and NYHA functional class at weeks 4 and 12, and 6-min walk distance, likewise showed a statistically significant improvement.

The benefit of FCM treatment in the primary endpoint was similar in patients with and without anemia.

After the publication of the FAIR HF trial, the 2012 ESC guidelines on HF (49) considered ID a relevant comorbidity that influences patient outcomes.

The FCM evaluatioN on perFormance in patients with IRon deficiency in coMbination with chronic Heart Failure (CONFIRM-HF) trial (50) was the second large-scale trial of FCM in HF. In this multicenter, randomized double-blind study, 304 patients with ejection fraction < 45% and elevated BNP levels were enrolled. Patients were randomized to FCM or placebo. The follow-up was 1 year (6 months longer than that in the previous FAIR-HF study). Participants who received FCM showed an improvement of functional capacity and quality of life and a decrease in hospitalization for worsening of HF, regardless of anemia. For the first time, functional capacity (the primary endpoint) was assessed with an objective test: the 6MWT. A significant difference in 6MWT at week 24 was observed in the FCMM group vs. the placebo group (difference FCM vs. placebo: 33 ± 11 m; *p* = 0.002).

The study confirmed the safety profile of FCM: there were no differences in investigator reported adverse events, serious adverse events and adverse event leading to a further update to the guidelines for HF in 2016 (5).

The Effect of ferric carboxymaltose on exercise capacity in patients with chronic heart failure and iron deficiency (EFFECT-HF) study (51) showed that FCM also improves the VO_2_ peak, as measured by CPET. In this randomized study, in a non-blinded fashion, 172 patients in NYHA class II or III, with ejection fraction <45% and a VO_2_ peak between 10 and 20 ml/kg/min, as measured by CPET, were enrolled and followed for 6 months. The primary endpoint was the improvement in the VO_2_ peak at the end of the study. This result has been documented in patients receiving EV iron, but it was not significant. The probable explanation for these results is a bias in patient selection (non-blinded study) leading to higher mortality in the control arm and therefore to a smaller amount of obtainable data.

To date, sufficient evidence indicates that the treatment of iron depleted chronic patients with HF and a reduced left ventricular ejection fraction with FCM is associated with improved functional capacity and quality of life.

On the basis of this evidence, the ESC guidelines recommend FCM for symptomatic patients with HF and a reduced ejection fraction with ID (defined by serum ferritin <100 μg/L, or ferritin between 100 and 299 μg/L and TSAT < 20%) to improve symptoms and functional capacity. This is a class IIa recommendation with a level of evidence of A (5).

Even if no clinical trials have been designed to demonstrate the benefit of iron therapy in patients with HF, in terms of decreases in hospitalization or other hard clinical endpoints, some promising preliminary data have been reported.

A possible clinical benefit has been suggested by a *post-hoc* sensitivity analysis of the CONFIRM trial. The composite risk of first hospitalization due to worsening HF or all-cause death has been found to be significantly lower in actively treated patients, with an HR of 0.53(0.30–0.95) *p* = 0.03 (50).

Two recent meta-analyses (51, 52) have shown that FCM significantly decreases hospitalization, cardiovascular and total mortality, and improves the functional class, exercise capacity and quality of life in patients with HF.

Approximately 800 patients treated with FCM had a lower rate of cardiovascular hospitalization and CV mortality (rate ratio 0.59) compared to control group. Treatment with FCM decreased the all-cause mortality (rate ratio 0.59) (51).

In an exploratory hypothesis generating analysis, Grote Beverborg et al. have evaluated the effect of FCB in the patients included in these meta-analyses, who were selected on the basis of a cutoff of TSAT ≤ 19.8% and serum iron ≤ 13 μmol/L. The authors found out that patients with a TSAT ≤ 19.8% had improved outcomes after treatment with FCM. In contrast, in patients with low ferritin but TSAT ≥ 19.8, FCM treatment did not improve outcomes (6).

### IV Iron in Acute HF: The AFFIRM-AHF Trial

The AFFIRM-AHF trial (a randomized, double-blind, placebo controlled trial comparing the effect of intravenous ferric carboxymaltose on hospitalizations and mortality in iron deficient subjects admitted for acute heart failure) is the first placebo-controlled trial the designed to evaluate the effect of FCM in patients hospitalized for acute HF (53).

The trial enrolled patients hospitalized for acute HF with an ejection fraction <50%. Most patients were in NYHA class II or III.

According to the definition of ID used in previous studies, patients were required to have a serum ferritin <100 μg/L, or 100–299 μg/L with TSAT < 20% to be eligible.

Patients were randomized to active treatment with FCM or placebo.

Patients in the active treatment group received the first dose of FCM shortly before discharge from the hospital and the second dose at week 6. Maintenance doses were given at weeks 12 and 24 if ID persisted. However, 80% of patients required only one or two infusions.

Among 1,525 patients, 567 were randomized to FCM. The primary endpoint of the study was a composite of HF hospitalizations and cardiovascular death during a 1 year follow-up.

The patients randomized to active treatment had a lower prevalence of the primary endpoint (293 vs. 372, RR 0.79, 95% CI 0.62–1.01), with marginal statistical significance (*p* = 0.059). Patients who were actively treated had significantly fewer hospitalizations for HF: rate ratio 0.79 (0.58–0.94, *p* = 0.013). The secondary endpoint, a composite of first HF hospitalization or cardiovascular death, was reduced hazard ratio 0.80 (066–0.98, *p* = 0.030).

Because the course of the trial was influenced by the COVID-19 pandemic, a sensitive analysis in which patients were censored in each country on the date when the first patients with COVID-19 were reported in the respective country, was performed. This analysis confirmed the significant decrease in of HF hospitalizations in treated patients, regardless of the presence of anemia.

This trial suggests several relevant clinical implications, most notably that ID is a modifiable risk factor for hospitalization. According to the AFFIRM-AHF trial, during the acute phase of the disease, when hospitalization is required, ID should be verified, (dosing ferritin and TSAT) and treated with FCM. Treatment should be started before discharge.

## Ongoing Studies

Some ongoing trials have been designed to explore the benefit of treatment of ID at different points during the trajectory of HF (54).

### FAIR-HFpEF

The Effect of IV Iron in Patients with HF With Preserved Ejection Fraction study addressed whether treatment with FCM for patients with HF with a preserved ejection fraction and ID might improve exercise capacity, and symptoms.

### HEART-FID

The Randomized Placebo-controlled Trial of FCM as Treatment for Heart Failure with Iron Deficiency trial is a double-blind, multicenter, prospective, randomized, placebo-controlled study designed to assess the effects of IV FCM compared with the placebo in terms of rate of death, hospitalization for worsening HF and 6-month change in 6MWT.

### FAIR-HF2

The Intravenous Iron in Patients with Systolic Heart Failure and Iron Deficiency to Improve Morbidity & Mortality trial was designed to assess whether long-term therapy with FCM compared with placebo might decrease the rate of recurrent HF hospitalizations and cardiovascular death in patients with HF with a reduced ejection fraction.

### IRONMAN

The Intravenous Iron Treatment in Patients With HF and ID study will address whether the additional use of intravenous iron administered as iron (III) isomaltoside 1,000 iron will improve clinical outcomes for patients with HF and ID.

## Conclusion

Several lines of evidence indicate that ID is a target for therapy for both acute and chronic HF.

Patients with symptomatic HF should be screened for ID during both the chronic stable phase than during acute decompensation requiring hospitalization.

**Figure 2:**
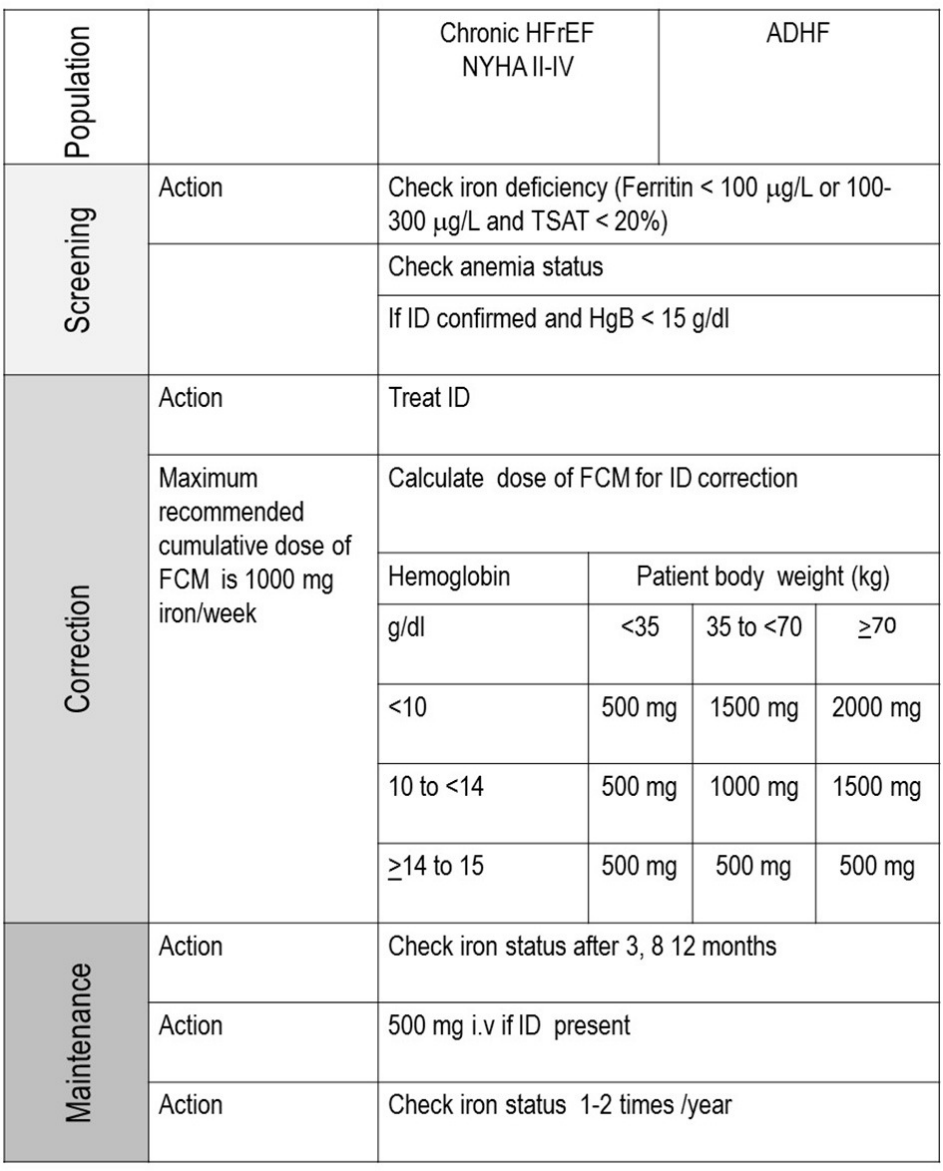
Algorithm for diagnosis and treatment of ID.

FCM infusion should be administered in patients with HF with a reduced ejection fraction in whom ID is confirmed, to improve functional capacity, and in patients with acute HF, to reduce re-hospitalization after discharge (Figure 2).

Ongoing trials will demonstrate whether treating ID may improve clinical outcomes in patients with chronic HF with both reduced and preserved ejection fractions.

## Author Contributions

AP and DS contributed to conception and design of the review. AP, CR, RR, and RC wrote sections of the manuscript. All authors contributed to manuscript revision, read, and approved the submitted version.

## Conflict of Interest

The authors declare that the research was conducted in the absence of any commercial or financial relationships that could be construed as a potential conflict of interest.

## Publisher's Note

All claims expressed in this article are solely those of the authors and do not necessarily represent those of their affiliated organizations, or those of the publisher, the editors and the reviewers. Any product that may be evaluated in this article, or claim that may be made by its manufacturer, is not guaranteed or endorsed by the publisher.
